# Connecting behaviour change to systems change: rethinking climate action in scientific societies

**DOI:** 10.1186/s12966-026-01983-0

**Published:** 2026-10-02

**Authors:** Loes Crielaard, Jillian O’Mara, Coosje Dijkstra, Wilma Waterlander

**Affiliations:** 1 Department of Public and Occupational Health, Amsterdam UMC, Location University of Amsterdam, Amsterdam, The Netherlands; 2 Health Behaviours and Chronic Diseases, Amsterdam Public Health Research Institute, Amsterdam, The Netherlands

**Keywords:** Climate change, Behaviour change, Systems change, Systems science, Conferences

## Abstract

Climate change is increasingly recognised as one of the greatest threats to public health, and scientific societies, like the International Society of Behavioral Nutrition and Physical Activity (ISBNPA), are rightly asking how they can contribute to meaningful action. Yet a fundamental question remains largely unanswered: how can individual action be connected to efforts to address systemic drivers? At its 2026 conference in Cádiz, Spain, ISBNPA asked presenters to declare the estimated carbon emissions from their travel to the conference. Well-intentioned as it is, viewed through a systems lens, such an intervention risks placing disproportionate emphasis on individual responsibility when it is not embedded within broader institutional and structural change. Here we see parallels with complex public health problems within ISBNPA’s scientific scope, where individual behaviours are increasingly understood as shaped by commercial, political and environmental forces. Building on the ISBNPA Climate Action Committee’s initiative to rethink conference travel and its 2026 commentary, we examine this issue through four systems science lenses – shifting the burden, the fundamental attribution error, pushing stones uphill and unintended consequences – and outline three opportunities to connect behaviour change to systems change within ISBNPA: conference paradigm, research priorities and collective advocacy.

Climate change is increasingly recognised as one of the greatest threats to public health [1]. Scientific societies, like the International Society of Behavioral Nutrition and Physical Activity (ISBNPA), are rightly asking how they can contribute to meaningful action [2]. Yet a fundamental question remains largely unanswered: how can individual action be connected to efforts to address systemic drivers? This question is not unique to climate change. It applies equally to complex public health problems that ISBNPA increasingly approaches from a systems perspective – e.g. obesogenic environments driven by commercial interests – which may well share systemic drivers with the climate crisis [1]. Climate action in scientific societies brings the question of where to intervene into sharp focus.

ISBNPA has embraced the ‘planetary health’ concept, which emphasises interdependence between human and environmental health [3]. In addition to including ‘planetary health’ in its mission, ISBNPA has formed the Climate Action Committee, which has pledged to cut the Society’s carbon footprint in half by 2030 [2].

This ambition cannot be reached by making fragmented changes here and there. At this year’s 2026 conference in Cádiz, Spain, the Committee therefore posed a deeper question to members [4]: given that 90% of ISBNPA’s footprint comes from conference travel, would you prefer to (a) reduce the frequency of global conferences and introduce regional meetings in the ‘off years’, or (b) revise our carbon reduction target?

Equally probing, presenters were asked to begin their presentations by declaring the estimated carbon emissions from their travel to Cádiz. Presentation templates also included a pre-written statement: “Commitment: Efforts were made to minimise environmental impact where feasible.”

This request gave many of us pause, not only because it confronted our choice to travel to the conference (*had* we made efforts to minimise environmental impact?), but also because something about it did not sit right.

How can individual action be connected to efforts to address the systemic drivers of the climate crisis, harnessing our influence as a scientific society? Building on the Climate Action Committee’s recognition in their 2026 commentary [2] that focusing on conferences alone is insufficient, we explore this question through four systems science lenses.

Connecting behaviour change to systems change would align ISBNPA’s climate action with its scientific scope. Systems science contributions reached a record level at the 2026 conference, with a dedicated track running throughout the programme. Systems approaches in public health treat today’s persistent problems as demanding systems change [5]: shifting thinking from individuals to systems, from cells to society, viewing (health-related) behaviour as the outcome of interacting societal causes – that could be disrupted to improve (planetary) health.

The request to declare our footprints has sparked discussion, and prompted this commentary. Yet well-intentioned as it is, viewed through a systems lens, such an intervention risks placing disproportionate emphasis on individual responsibility when it is not embedded within broader institutional and structural change. It may thereby divert attention from higher leverage action and produce unintended consequences.

## Systems lens 1: shifting the burden

**Fig. 1:**
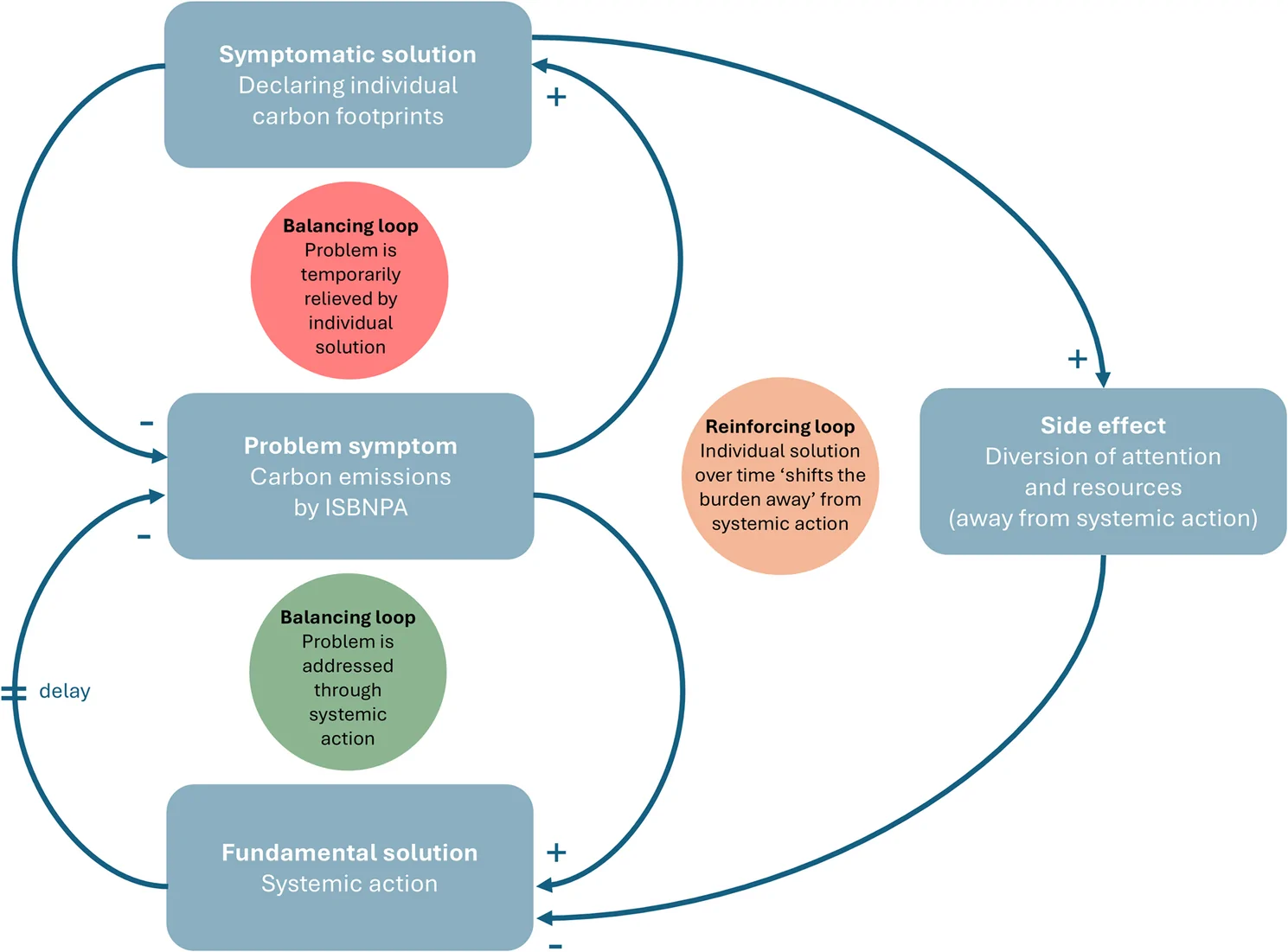
The individual solution, declaring individual carbon footprints, may over time ‘shift the burden’ away from systemic action. ‘Shifting the burden’ is a standard system structure in the system dynamics literature [6], comprising a problem symptom, a symptomatic solution, a side effect and a fundamental solution, together with the links and loops connecting them. Here it is applied to the declaration of individual carbon footprints. Plus: a change in factor 1 causes a change in factor 2 in the *same* direction. Minus: a change in factor 1 causes a change in factor 2 in the *opposite* direction. The double line mark on the arrow indicates a delay

In system dynamics, this diverted attention is a well-recognised system structure, known as ‘shifting the burden’ [6] (Fig. 1). A symptomatic solution – declaring footprints – may temporarily relieve the problem symptom – ISBNPA’s emissions – through peer pressure or shame, leading some members to choose alternative travel, or even not attend the conference. The problem is not the symptomatic solution itself, but its potential to substitute for or divert attention from more fundamental action.

The side effect is that this ‘quick fix’ may divert attention and resources away from the fundamental solution. This occurs either because we (inadvertently) think we are already doing enough by declaring our footprints, or because overemphasising individual responsibility paralyses us – in a ‘wrong’ system, we are all ‘wrong’ – and we end up doing nothing.

Consider another unintended consequence of attention being diverted: who benefits when we point to one another rather than to the system? Not coincidentally, the framing of climate change as an individual responsibility appears to have been popularised by the fossil fuel industry, who coined the ‘carbon footprint’ concept [7].

## Systems lens 2: the fundamental attribution error

Meadows’ Places to Intervene in a System [8] identifies paradigms, the mental models from which systems arise, as the highest leverage point for systems change, while individual-focused interventions rank lowest. Sterman calls this the fundamental attribution error: “the tendency to blame the person rather than the system” [9]. Such responses leave the dynamics that led to the outcome untouched, treating people as isolated elements rather than parts of a broader system, likely pushing the next person toward the same outcome.

Footprints of individual delegates may reflect little more than ISBNPA’s location in a given year. Declaring them does not alter the dynamics that make us want to attend the conference.

## Systems lens 3: pushing stones uphill

We see parallels with public health debates, where complex problems like the global rise in obesity are often framed as individual responsibility (“humans [must have] collectively lost willpower around the 1980s” [10]), rather than as products of the environments, policies and markets that shape behaviour [1].

The metaphor of pushing a stone uphill captures this well. Consider the effort required from individuals to make healthier or more sustainable choices, while the system continuously pushes them in the opposite direction. Individual behaviour change interventions depend on constant reinforcement as they do not alter the conditions generating the problem. Reshaping the hill itself, through changes in incentives, infrastructures and industries, can make healthy, sustainable choices the default.

## Systems lens 4: unintended consequences

What choices do we each have if we leave our system unchanged? The footprint declaration is well meant, but followed to its conclusion, the unintended consequence is that the researchers who contributed the most were those who did not attend ISBNPA at all. Their contribution goes unrecorded – there is no slide on which to declare it.

Within the current system, can we choose not to attend? As the Climate Action Committee notes, conference travel is not a luxury for some but vital to career-building [2]. The unintended consequence we observed was early-career researchers, who already carry a disproportionate share of climate anxiety, expressing guilt about participating in an event from which they stood to gain most.

Can we, individually, choose not to fly? Although Meadows considers individual behaviour change the lowest leverage point, she notes this shifts once a critical mass is reached [8]. Credit is due to those who travelled by other means. The unintended consequence, however, is narrowing who can attend and how they attend, by geographical location, time, money, physical capability and family circumstances.

Can we, as a Society, choose not to hold conferences? The annual conference underpins the Society’s economic feasibility [2]. Online formats attract less funding and are difficult to run across time zones. The unintended consequence is a threat to ISBNPA’s viability.

## Connecting behaviour change to systems change

These four lenses do not imply that individual action is irrelevant. System dynamics, with its focus on feedback loops, holds that structure and agency are mutually dependent. Structures constrain individual actions, but are also created, perpetuated and changed by them. The question is not whether individual action matters, but whether it is organised to reproduce existing structures or transform them. For ISBNPA, we see three opportunities to connect behaviour change to systems change.

### Conference paradigm

A first step is asking what mental model underpins conferences at ISBNPA. What is keeping current practice in place? What do we actually value about conferences, if we could decide all over again? Alternating between global conferences and regional meetings – as the Climate Action Committee proposed and 301/393 delegates voted for when confronted with the dilemma [4] – alongside hybrid formats could be part of the solution. More fundamentally, decisions should be driven by reconsidering when in-person attendance is necessary and when other forms of international collaboration may be appropriate. In-person attendance could for example be more impactful if we use face-to-face time to work together, rather than present research. ISBNPA could position itself as a frontrunner in the scientific community - as it has for systems science - by sharing not only its final decisions, but also the assumptions that needed to be reconsidered, paving the way for a new conference paradigm. The Committee set a precedent for transparency, writing openly about dilemmas in this journal [2].

### Research priorities

The research we conduct is where we, a group of researchers, can make the greatest impact (conform ISBNPA’s By-Laws [3] and as argued by the Climate Action Committee [2]). Much of it focuses on changing the behaviour of citizens, patients or consumers. Yet some of the greatest opportunities may lie upstream, in influencing the behaviour of governments, corporations, investors and urban planners – who shape the systems within which individual choices are made, whether for energy consumption, ultra-processed food intake or social media habits. ISBNPA could support this shift by making systemic drivers a research priority.

### Collective advocacy

ISBNPA, as a collective, a critical mass of researchers, has the potential to create far greater impact than any of its members could achieve alone [2]. The time we spent calculating our footprints – minutes each, weeks together – shows what we can mobilise. What if that same capacity went toward the systems, policies and incentives that shape these choices? One concrete step would be to broaden the mandate of the Climate Action Committee towards systems-level climate and health action. Such a group could connect the three areas outlined here: supporting continued development of a sustainable and equitable conference model, identifying and promoting systems-oriented research priorities and coordinating advocacy beyond the Society. Internally, this could mean advocating for changes to academic incentives that currently shape researchers’ choices. Externally, this could mean drawing on our position and influence as researchers – through lobbying, agenda-setting, political engagement and collaboration with policymakers – to ensure the right evidence reaches the right people at the right time, to change the systems we study.

## Conclusions

These four lenses do not imply that individual action is irrelevant. Structures constrain individual actions, but are also created, perpetuated and changed by them. The question is not whether individual action matters, but whether it is organised to reproduce existing structures or transform them. The time we spent calculating our carbon footprints at ISBNPA – minutes each, weeks together – shows what we can mobilise. What if that same capacity went toward the environments, policies and markets within which individual choices are made? Connecting behaviour change to systems change would align ISBNPA’s climate action with its scientific scope – as a frontrunner in systems science – and draw on our position and influence as researchers to change the systems we study.

## Data Availability

No datasets were generated or analysed during the current study.

## Abbreviations

ISBNPA: International Society for Behavioral Nutrition and Physical Activity
